# The Mcu1 mitochondrial protein coordinates TCA cycle enzymes to modulate phenotypic switching and commensalism in *Candida albicans*

**DOI:** 10.1080/21505594.2026.2711487

**Published:** 2026-08-02

**Authors:** Mingyang Ma, Ming Xu, Shuyun Guan, Shuru Fan, Guanghua Huang, Li Tao

**Affiliations:** a State Key Laboratory of Genetics and Development of Complex Phenotypes, School of Life Sciences, Shanghai Institute of Infectious Disease and Biosecurity, Department of Laboratory Medicine, Department of Infectious Diseases, Huashan Hospital, Fudan University, Shanghai, China; b State Key Laboratory of Microbial Resources, Institute of Microbiology, Chinese Academy of Sciences, Beijing, China

**Keywords:** *Candida albicans*, commensalism, white-to-opaque switching, Mcu1, TCA cycle enzymes, Wor1

## Abstract

*Candida albicans* is a common resident of humans that colonizes multiple sites in the human body, such as the gut, in healthy individuals. In immunocompromised hosts, however, it can switch to a pathogenic state and cause infections. The molecular mechanisms underlying this commensal-pathogenic transition have not been fully elucidated. Here, we demonstrate that the mitochondrial protein Mcu1, which is required for utilization of multiple carbon sources, plays a crucial role in N-acetylglucosamine (GlcNAc)-induced phenotypic switching and gut commensalism in *C. albicans*. Disruption of Mcu1 or key TCA cycle enzymes impaired GlcNAc utilization, blocked white-to-opaque switching under *in vitro* culture conditions, and reduced gut colonization in a murine model. Mechanistically, Mcu1 sustains respiratory metabolism by regulating key oxidoreductases, while also promoting gut commensalism by enabling *in vivo* activation of the master regulator Wor1. Collectively, our findings reveal that Mcu1 and key TCA cycle enzymes play an essential role in phenotypic switching and cooperatively regulate the commensal-pathogenic transition in *C. albicans*.

## Introduction

*Candida albicans* is the predominant causative agent of life-threatening invasive fungal infections [1]. Paradoxically, this fungus primarily exists as a commensal colonizer of the healthy human gastrointestinal tract [2]. Cumulative evidence indicates that disseminated *C. albicans* infections frequently originate from patients’ own endogenous commensal strains [3,4]. This commensal-pathogen duality is tightly linked to the white-to-GUT (W-G) switching, a global reprogramming of cellular identity that underlies *C. albicans*’ transition from commensalism to pathogenesis [5].

The W-G switching, which occurs exclusively within the host gut, is analogous to the well-characterized *in vitro* white-to-opaque (W-O) switching and exemplifies a typical morphological transition system in *C. albicans* [5]. White, opaque, and GUT cell types exhibit distinct morphological and pathogenic characteristics [5–7]. White cells are small and round, suited for bloodstream dissemination and systemic infection. Opaque cells appear larger and elongated, displaying an enhanced capacity for superficial infection. GUT cells resemble opaque cells morphologically but represent a specialized commensal form adapted to the intestinal environment. Despite morphological similarities between GUT and opaque cells, they differ in several critical aspects: (1) *MTL* configuration; (2) temperature sensitivity; (3) cell surface architecture (e.g. the presence of “pimple” structures); (4) mating competence; (5) transcriptomic profiles [5].

The regulation of W-G switching shares core mechanisms with W-O switching [5]. Both phenotypic transitions can be induced by specific environmental cues including CO_2_ and N-acetylglucosamine (GlcNAc). Wor1 serves as the master regulator for both W-G and W-O switching, and is essential for the formation of both GUT and opaque cell types. *In vitro*, *WOR1* expression is repressed by the **a**1-α2 heterodimer, such that only *MTL* homozygous strains can derepress *WOR1* to promote opaque cell formation. Within the gastrointestinal tract, however, distinct mechanisms prevail: host-derived signals, such as GlcNAc, can promote the accumulation of *WOR1* transcript and trigger and maintain the GUT cell state even in *MTL-*heterozygous (**a**/α) strains [5]. Notably, GlcNAc is a major component of mucosal tissue and bacterial cell walls, serving both as a nutrient source and a crucial signal for phenotypic switching and commensalism in *C. albicans* [8].

Environmental adaptation, morphological plasticity, and pathogenicity in *C. albicans* are tightly linked to its metabolic flexibility. The three cell types exhibit markedly distinct metabolic profiles: white cells favor fermentative metabolism; opaque cells rely more on aerobic respiration; and GUT cells preferentially utilize GlcNAc [5,9]. Consistently, opaque cells upregulate genes involved in glycolysis, the tricarboxylic acid (TCA) cycle, and the glyoxylate cycle. In contrast, GUT cells enhance the expression of key GlcNAc utilization components together with TCA cycle genes, supporting their commensal fitness in the gastrointestinal tract. These findings suggest that TCA cycle enzymes play a crucial role in sensing intestinal GlcNAc to regulate the switch between commensal and pathogenic states. Consistent with this, our prior work identified the mitochondrial protein Mcu1 as a specific sensor for GlcNAc, which is essential for regulating morphological switching and virulence in *C. albicans* [10]. In *C. auris*, Mcu1 similarly regulates oxidative metabolism, thereby facilitating filamentous growth and skin colonization [11]. Despite these advances, the molecular mechanisms by which Mcu1 and the TCA cycle modulate the transition between commensalism and pathogenicity remain poorly understood.

In this study, we demonstrate that the mitochondrial protein Mcu1, in coordination with TCA cycle enzymes, regulates phenotypic switching and gut commensalism in *C. albicans*. Mechanistically, Mcu1 modulates respiratory metabolism by regulating the expression of a subset of oxidoreductases and facilitates gut commensalism by activating the master regulator Wor1 under host conditions. Taken together, our findings indicate that Mcu1 and the TCA cycle cooperatively govern the commensal-pathogenic transition of *C. albicans*.

## Materials and methods

### Strains and growth media

All *C. albicans* strains used in this work were derived from the wild-type (WT) reference strain SC5314 and are presented in Table S1. Strains were recovered from −80°C glycerol stocks and routinely cultured on yeast extract-peptone-dextrose (YPD) medium (20 g/L peptone, 10 g/L yeast extract, 20 g/L glucose) at 30°C. To prepare solid media, 2% (w/v) agar was incorporated into the liquid medium. Yeast extract (BD Difco 212750), peptone (BD Difco 211677), and agar (BD Difco 214010) were obtained from BD Biosciences (Sparks, MD), and glucose (G8270) was from Sigma-Aldrich (St. Louis, MO). Lee’s glucose (2%, w/v) and Lee’s glucose (0.5%, w/v) +GlcNAc (1.5%, w/v) media were employed for the white-to-opaque (W-O) switching assays [12]. To facilitate phenotypic discrimination between white and opaque colonies, the dye phloxine B (5 µg/mL, P2759, Sigma-Aldrich) was added to the medium. Opaque colonies uptake the dye and appear pink/red, whereas white colonies remain unstained. Deletion mutants and *WOR1*_*prom*_*-FLP* strains were selected and maintained on YPD medium containing nourseothricin, on synthetic complete dextrose (SCD) medium, or on 5-fluoroorotic acid (5-FOA) medium, as appropriate [5].

### Construction of plasmids and *C.* *albicans* strains

The primers used for PCR amplification in this study are presented in Table S2. To construct plasmid *pACTS-WOR1*, the *WOR1* gene was amplified by PCR from *C. albicans* SC5314 genomic DNA and then cloned into plasmid *pACTS* [13] using the *Eco*RV/*Hind*III restriction sites. To achieve ectopic *WOR1* expression, the *Asc*I-linearized *pACTS-WOR1* plasmid was transformed into strain GH1013 or the *mcu1/mcu1* mutant.

To delete the *MCU1* gene in the strain GH1013 or SN1020, a fusion PCR-based strategy was employed [14]. Briefly, the 5’ and 3’ flanking fragments of the *MCU1* locus were PCR-amplified from *C. albicans* SC5314 genomic DNA with primer pairs MCU1up-Fwd/MCU1up-Rev and MCU1down-Fwd/MCU1down-Rev, respectively. The selectable marker genes (*CdARG4* and *CdHIS1*) were amplified separately from plasmids pSN69 and pSN52 [14]. Two deletion cassettes were then constructed by fusion PCR, each consisting of a selectable marker (*CdARG4* or *CdHIS1*) flanked by the homologous 5’ and 3’ regions of *MCU1*. These cassettes were sequentially introduced into strain GH1013 or SN1020 [5].

All *MTL* homozygous strains were constructed by transforming the corresponding *MTL* heterozygous parental strains with the *Apa*I*/Sac*I-linearized plasmid pSFS2A-L23.14, resulting in the stochastic deletion of either the *MTL***a** or *MTL*α allele [15].

### White-to-opaque (W-O) switching assays

White-to-opaque (W-O) switching assays were performed as previously described, with minor modifications [15]. Briefly, white cells from five-day-old cultures were replated on Lee’s media and incubated at 25°C for 5 or 10 days under either ambient air or 5% CO_2_. The W-O switching frequency was calculated using the following formula: [(number of opaque colonies + sectored colonies) / (total number of colonies)] × 100%. To confirm colony phenotypes, cellular morphology was examined in representative colonies of each type. All isolates used for subsequent experiments were derived from single-colony purifications.

### Proteomic analysis

For proteomic analysis, four biological replicates of each strain were grown overnight in YPD medium at 30°C, then sub-cultured into fresh YPD and harvested at mid-log phase. Total protein was extracted as previously described with minor modifications [11]. Briefly, harvested cells were washed twice with ice-cold 1×PBS and resuspended in 200 μL of lysis buffer (50 mM Tris-HCl, pH 8.0, 150 mM NaCl, 1% sodium deoxycholate, 1% NP-40, 0.1% SDS, 1 mM EDTA, 1 mM EGTA, 1 mM PMSF) supplemented with a protease inhibitor cocktail (Cat. No. 11873580001, Roche Diagnostics). Cell lysis was performed by bead beating with eight cycles (40 s of beating, alternating with 1 min of cooling on ice). Protein lysates were clarified by centrifugation, and protein concentration was examined using Bradford assay (Sigma-Aldrich).

Liquid chromatography-mass spectrometry (LC-MS/MS) analysis was performed in a label-free quantitative (LFQ) mode, providing relative quantification of protein abundance between the WT and *mcu1/mcu1* mutant [11]. Total proteins were digested using the Filter-Aided Sample Preparation (FASP) protocol with Nanosep 10k filters (Pall Life Science) [16]. Briefly, samples were subjected to three rounds of buffer exchange with 8 M urea in 25 mM NH_4_HCO_3_, followed by reduction with 10 mM dithiothreitol (DTT) and alkylation with 30 mM iodoacetamide. The filters were subsequently washed once with 20% acetonitrile (ACN) and three times with digestion buffer (30 mM NH_4_HCO_3_). Trypsin digestion was then performed overnight at an enzyme-to-protein ratio of 1:50 (w/w). The resulting peptides were collected by filtration, and the filters were washed twice with 15% ACN. The filtrates were combined, vacuum-dried, and then subjected to LC-MS/MS analysis on an EASYnLC 1200 system (Thermo Fisher Scientific), which was coupled to a timsTOF HT mass spectrometer (Thermo Fisher Scientific). Separation was achieved on a home-packed C18 column (75 μm i.d. ×25 cm; packed with ReproSil-Pur 120 C18-AQ, 1.9 μm particles, Dr. Maisch GmbH) in a one-column configuration [17]. The LC mobile phases comprised Solvent A (0.1% formic acid in water) and Solvent B (0.1% formic acid in 80% ACN). Eluted peptides were directly introduced into the timsTOF HT mass spectrometer. Mass spectrometry data were acquired in a data-dependent acquisition (DDA) mode. Full-scan MS1 spectra (m/z 350–1600) were collected in the Orbitrap at a resolution of 60,000 with automatic maximum injection time control. The duty cycle time was set to 3 s. Following higher-energy collisional dissociation (HCD) fragmentation (30% normalized collision energy), MS2 spectra were acquired in the Orbitrap analyzer at a resolution of 15,000.

Raw data were analyzed with Proteome Discoverer software (v2.4, Thermo Fisher Scientific) and queried against the *C. albicans* protein database (downloaded from UniProt) using the Mascot search engine (v2.7.0, Matrix Science). Database searches were conducted with the following parameters: trypsin/P was specified as the protease with up to two missed cleavages permitted; precursor mass tolerance was 10 ppm and fragment mass tolerance was 0.05 Da; carbamidomethylation was specified as a fixed modification on cysteine residues; and oxidation of methionine and N-terminal acetylation were included as variable modifications. The false discovery rate (FDR) thresholds were set at 0.05 for proteins and 0.01 for peptides. Four biological replicates were carried out. Differentially expressed proteins were identified employing the DEP package (v3.16) in R, applying a dual-filter criterion of |Log_2_(fold change)| ≥ 1 and adjusted *p*-value < 0.05 [11,18]. Protein IDs were mapped to official gene names using the Candida Genome Database (CGD) and UniProt. Gene Ontology (GO) enrichment was carried out as an over-representation analysis (ORA) using the GO resource (https://www.geneontology.org/), with the set of DEPs as input. Statistical significance was evaluated by Fisher’s exact test and corrected for multiple testing using the Benjamini–Hochberg procedure; GO terms with adjusted *p*-value < 0.05 were considered significantly enriched. Key findings were visualized using the online platform, Chiplot (https://www.chiplot.online/).

### Animal experiments

All animal experiments were conducted under a protocol approved by the Animal Care and Use Committee of Fudan University (2021JS0012), and were conducted in accordance with the committee’s ethical guidelines. All experimental procedures were carried out in compliance with the ARRIVE guidelines. Specific-pathogen-free (SPF) female BALB/c mice (6 weeks old, 18–20 g) were purchased from Beijing Vital River Laboratory Animal Technology Co., Ltd and used for all experiments. Mice were routinely housed under SPF conditions at 21°C, 50–70% relative humidity, with a 12-h light/dark cycle.

To assess the gut colonization capacity of the WT, mutant, and reconstituted strains, a total of 27 female BALB/c mice (6 weeks old) were randomly group-housed (*n* = 3 per cage) under standard controlled conditions. To facilitate colonization, mice were pre-treated with antibiotics (1 mg/mL each of ampicillin, kanamycin, chloramphenicol, and streptomycin) in their drinking water for 3 days. Mice were then inoculated via oral gavage with 5 × 10^7^ *C. albicans* cells in 200 µL of 1×PBS buffer. Starting 3 days post-inoculation, fecal pellets were collected, homogenized, and serially diluted. Samples were plated on YPD agar for colony-forming unit (CFU) counting. Gut colonization levels are expressed as log_2_(CFU per gram of feces or cecal tissue). The colonization experiment was terminated on day 21 post-gavage. Following anesthesia, mice were euthanized by cervical dislocation. The euthanasia procedures were performed in compliance with the American Veterinary Medical Association guidelines. Tissue samples from the cecum were collected and plated on YPD agar for CFU counting.

The frequency of *WOR1* expression was quantified using a *WOR1*_*prom*_*-FLP* reporter system reported by a previous study [5]. For *in vitro* assay, reporter strains (WT and mutant) were serially passaged in liquid SCD medium at 30°C for eight generations, maintaining logarithmic growth. Cells were then plated on nonselective SCD medium and selective 5-FOA medium (with 25 μg/mL uridine, for selecting uracil auxotrophs). To confirm Flp-mediated recombination, a 346 bp fragment was amplified from the relevant strains using primers SNO509 and SNO840. The frequency of *WOR1*-expressing cells was estimated as: (number of 5-FOA-resistant colonies [confirmed by PCR]) / (total colony number) × 100%.

For *in vivo* model, a total of eight female BALB/c mice (6 weeks old) were randomly assigned to two groups (*n* = 4 per cage) and inoculated via oral gavage with reporter strains (WT and mutants), respectively. To facilitate colonization, mice were pre-treated with antibiotics (1 mg/mL each of ampicillin, kanamycin, chloramphenicol, and streptomycin) in their drinking water for 3 days. Mice were then inoculated via oral gavage with 1 × 10^8^ *C. albicans* cells in 200 µL of 1×PBS buffer. After 3 days, fecal pellets and cecal contents were collected, homogenized, and serially diluted. Samples were plated on YPD agar to assess colonization and SCD/5-FOA agar to determine switching frequency. The frequency was calculated as described above.

### Oxygen consumption rates (OCR) assays

Oxygen consumption rates (OCR) were determined using a Seahorse XFe96 Analyzer (Agilent) [19]. Data were processed with Wave software (v2.6). *C. albicans* cells were cultured to mid-log phase in liquid YPD medium, washed, and seeded at 5 × 10^4^ cells per well into Seahorse XF96 cell culture microplates (Agilent) pre-coated with 0.03% (w/v) poly-L-lysine. The plate was centrifuged at 500 × g for 3 min and incubated at 30°C for 1 h to facilitate cell adhesion. The XF Cell Mito Stress Test was performed in RPMI assay medium containing 10 mM glucose, 1 mM sodium pyruvate, and 2 mM glutamine. OCR was determined following sequential administration of mitochondrial inhibitors to final concentrations of: 100 µM dicyclohexylcarbodiimide (DCCD), 2 µM Carbonyl cyanide 4-(trifluoromethoxy) phenylhydrazone (FCCP), and 0.5 µM rotenone/antimycin A (Rot/AA). The key mitochondrial parameters – basal, ATP-linked, and maximal respiratory capacity were calculated from the acquired OCR data.

### Statistical analysis

Data are presented as mean ± standard deviation (SD) from at least three independent experiments. Statistical analyses were performed with GraphPad Prism. For comparisons, Student’s *t*-test (two groups) and one-way/two-way analysis of variance (ANOVA) with post-hoc testing (multi-groups) were applied. A *p*-value < 0.05 was considered statistically significant.

## Results

### Mcu1p is essential for GlcNAc-induced white-to-opaque (W-O) switching in *C.* *albicans*

**Figure 1.**
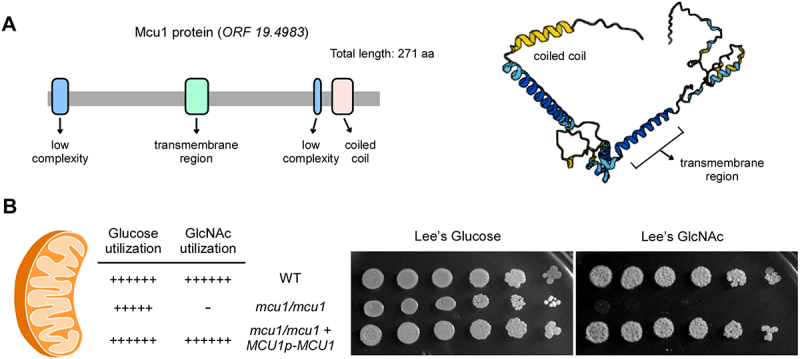
The mitochondrial protein Mcu1 is essential for GlcNAc utilization in *C. albicans*.

The host intestine is the primary niche for *C. albicans* colonization [20]. Within this niche, N-acetylglucosamine (GlcNAc), a constituent of mucosal tissue and bacterial cell walls, serves both as a nutrient source and a critical signal for morphological switching and commensalism [8]. We previously reported that Mcu1 (Multiple Carbon source Utilizer 1), a mitochondrial protein, is essential for GlcNAc utilization [10]. As shown in Figure 1(A) and in our previous study, Mcu1 is a 271-amino-acid protein with one predicted transmembrane domain, one coiled-coil domain, and two low-complexity regions (www.candidagenome.org). Deletion of *MCU1* completely abolished fungal growth on Lee’s GlcNAc medium and partially impaired growth on Lee’s glucose medium. Genetic complementation fully restored growth to wild-type (WT) levels (Figure 1(B)). These results demonstrate that Mcu1 is essential for GlcNAc utilization in *C. albicans*.

(A) Schematic diagram and predicted structure of *C. albicans* Mcu1 protein (encoded by *ORF 19.4983*). The protein is 271 amino acids long and contains one transmembrane domain, one coiled coil domain, and two low complexity domains. Domains and structure of Mcu1 protein were estimated using AlphaFold3 (https://www.alphafold.ebi.ac.uk/). (B) Growth of the WT (**a**/α), *mcu1/mcu1* (**a**/α) mutant, and the reconstituted strains (**a**/α) on Lee’s glucose and Lee’s GlcNAc medium plates. Different strains were initially grown in YPD liquid medium overnight at 30°C. Then, cells of each strain were collected, washed, and adjusted to 1 × 10^8^ cells/mL. Ten-fold serial dilutions were spotted onto Lee’s glucose and Lee’s GlcNAc medium plates, respectively, and cultured at 37°C for 3 days. WT, SC5314. The number of “+” signs indicates the degree of cell growth. “-” indicates that no growth was observed.

**Figure 2.**
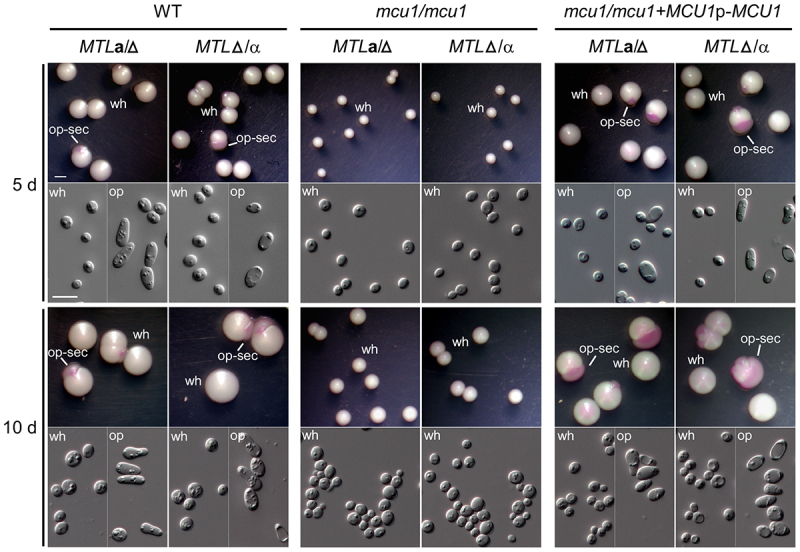
Deletion of *MCU1* blocks GlcNAc-induced white-to-opaque switching in *C. albicans*.

GlcNAc is one of the most potent environmental cues that induce W-O switching, a typical phenotypic transition linked to pathogenesis and host adaptation in *C. albicans* [12]. We therefore investigated whether Mcu1 is required for this process. Since the *mcu1/mcu1* mutant is unable to grow on media with GlcNAc as a sole carbon source, W-O switching was assessed on Lee’s medium containing a low concentration of glucose (0.5%, w/v) supplemented with GlcNAc (1.5%, w/v). The WT, *mcu1/mcu1* mutant, and reconstituted strains were constructed in both *MTL* homozygous (**a**/Δ and Δ/α) backgrounds, and the corresponding W-O switching was examined after 5 or 10 days of incubation at 25°C. As shown in Figure 2 and Table 1, although the W-O switching frequencies were 29.5 ± 2.9% and 42.3 ± 8.2% in the WT and reconstituted strains of *MTL***a**/Δ background, and 67.6 ± 9.8% and 48.4 ± 7.3% in those of *MTL*Δ/α background, no W-O switching was observed in the *mcu1/mcu1* (**a**/Δ or Δ/α) mutant strains. Moreover, low baseline W-O switching was observed even in the WT and reconstituted strains in the absence of GlcNAc (on Lee’s glucose [2%, w/v] medium) (Figure S1A and Table S3). Notably, exposure to 5% CO_2_, an additional environmental inducer of W-O switching, elicited near-complete switching frequency across WT, *mcu1/mcu1* mutant, and reconstituted strains (Figure S1B and Table S3). These results indicate that disruption of *MCU1* specifically abrogates GlcNAc-induced W-O switching but has no effect on CO_2_-induced W-O switching.

The WT (**a**/Δ or Δ/α), *mcu1/mcu1* mutant (**a**/Δ or Δ/α), and reconstituted strains (**a**/Δ or Δ/α) were initially grown on Lee’s glucose medium at 25°C for 5 days. White cells of each strain were replated onto Lee’s glucose (0.5%) + GlcNAc (1.5%) medium containing phloxine B and incubated at 25°C for 5 or 10 days. WT, SC5314. Wh, white; Op, opaque; Op-sec, opaque sector. Scale bar for colonies, 1 mm. Scale bar for cells, 10 μm. The numerical data are shown in Table 1.

**Table 1.** White-to-opaque switching frequencies of the WT, *mcu1/mcu1* mutant, and reconstituted strains on Lee’s glucose (0.5%) + GlcNAc (1.5%) medium at 25°C for 5 days.

Strain	Total colonies	% op colonies
WT (SC5314) (**a**/Δ)	291	29.5 ± 2.9
*mcu1/mcu1* (**a**/Δ)	205	<0.5
*mcu1/mcu1*+*MCU1*p-*MCU1* (**a**/Δ)	409	42.3 ± 8.2
WT (SC5314) (Δ/α)	509	67.6 ± 9.8
*mcu1/mcu1* (Δ/α)	327	<0.3
*mcu1/mcu1*+*MCU1*p-*MCU1* (Δ/α)	484	48.4 ± 7.3

### Mcu1 sustains respiratory metabolism by modulating the expression of oxidoreductases

**Figure 3.**
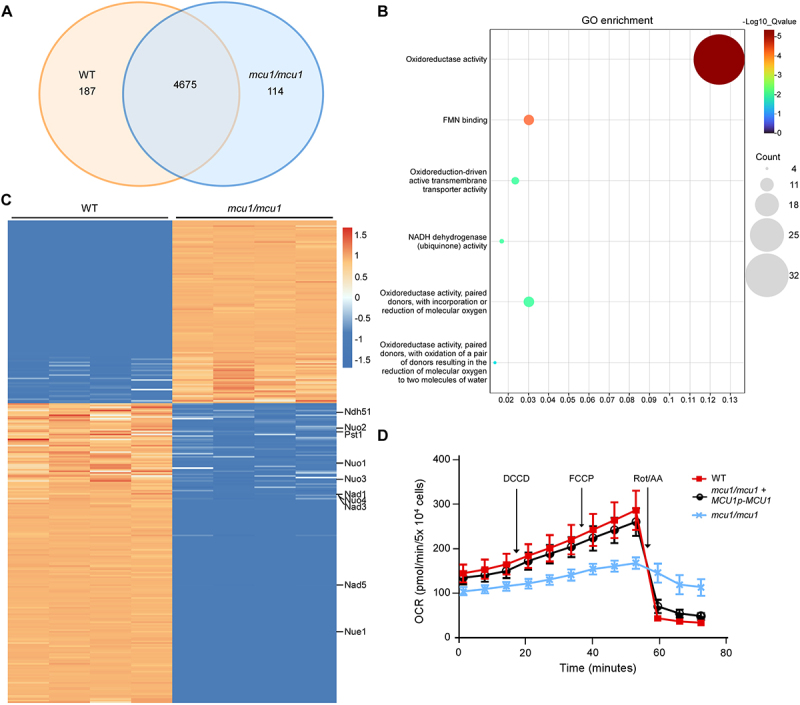
Deletion of *MCU1* affects respiratory metabolism by impairing the regulation of oxidoreductases.

To define the underlying mechanisms, we performed a comparative proteomic analysis of WT, *mcu1/mcu1* mutant, and reconstituted strains grown under GlcNAc-inducing conditions (Figure 3). In total, 301 proteins exhibited significant changes (|log_2_(fold change)| ≥ 1, *p* < 0.05), with 187 downregulated and 114 upregulated in the *mcu1/mcu1* mutant compared to WT (Figure 3(A)). Gene Ontology (GO) analysis was subsequently performed to investigate the functions of the differentially expressed proteins. Notably, a number of proteins with oxidoreductase activity were selectively diminished in the *mcu1/mcu1* mutant (Figure 3(B)). Prominent reductions were observed in mitochondrial respiratory chain-associated proteins, including multiple oxidoreductases such as Ndh51, Nuo1/2/3/4, Pst1, and Nad1/3/5 (Figure 3(C)). Based on these findings, the mitochondrial basal oxygen consumption rate (OCR) of the *mcu1/mcu1* mutant and WT strain was examined. As shown in Figure 3(D), intracellular OCR was significantly decreased in the *mcu1/mcu1* mutant compared to the WT and reconstituted strains, confirming impaired respiratory metabolism. This respiratory defect likely accounts for the mutant’s inability to grow on GlcNAc as a sole carbon source, as respiratory metabolism is required for energy production. Taken together, our findings suggest that Mcu1 plays a critical role in modulating respiratory metabolism by regulating the expression of a subset of oxidoreductases.

*C. albicans* cells were grown in YPD liquid medium at 30°C to log phase. WT (a/α), SC5314. (A) Venn diagram showing the differentially expressed proteins in *mcu1/mcu1* (a/α). (B) The Gene Ontology (GO) analysis of the differentially expressed proteins in *mcu1/mcu1* (**a**/α). Over-representation analysis (ORA) was carried out using the GO resource (https://www.geneontology.org/). Statistical significance was assessed by Fisher’s exact test and corrected for multiple testing using the Benjamini–Hochberg procedure. Only GO terms with an adjusted *p*-value < 0.05 are shown. Circle size is proportional to the number of differentially expressed proteins. The color of the bubbles represents the significance of the change in protein abundance (log_10_(adjusted *p*-value)). Protein categories (based on the GO analysis; Dataset S1). (C) The heatmap shows changes in the expression of proteins in *mcu1/mcu1* (**a**/α) relative to WT cells, |log_2_(fold change)| ≥1 and adjusted *p*-value < 0.05. The pheatmap R package (version 1.0.12; https://cran.r-project.org/web/packages/pheatmap/index.html) was used to plot expression levels of differentially expressed proteins. Proteins involved in oxidoreductase activity and respiratory metabolism are indicated. Colors represent the relative expression levels of proteins. The four columns for each strain represent four biological replicates. Detailed protein expression data are shown in Dataset S1. (D) OCR in the WT (**a**/α) and *mcu1/mcu1* (**a**/α) mutant strains was measured by a Seahorse XFe96 analyzer. Dicyclohexylcarbodiimide (DCCD, 100 µM); Carbonyl cyanide 4-(trifluoromethoxy) phenylhydrazone (FCCP, 2 µM); Rotenone/antimycin A (Rot/AA, 0.5 µM).

### Roles of TCA cycle enzymes in GlcNAc utilization and white-to-opaque (W-O) switching

**Figure 4.**
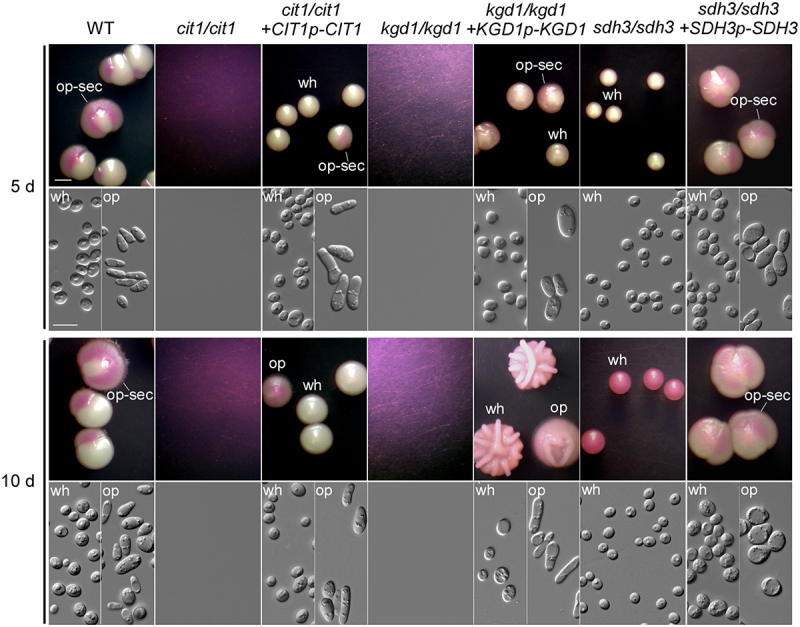
Effects of TCA cycle enzymes (Cit1, Kgd1, and Sdh3) dysfunction on GlcNAc-induced white-to-opaque switching in *C. albicans*.

Elevated respiratory metabolism is a critical feature of opaque cells in *C. albicans* [9]. As Mcu1 has been previously reported to physically interact with multiple TCA cycle enzymes (e.g. Cit1, Kgd1, etc.), we hypothesized that core TCA cycle components may regulate the utilization of GlcNAc and W-O switching [10]. To test this, we constructed the *MTL* homozygous mutants *cit1/cit1*, *kgd1/kgd1*, and *sdh3/sdh3*, and assessed growth and phenotypic switching in the presence of GlcNAc. Based on the results from Figure 2 and Table 1, which showed no difference of *MTL* type (**a**/Δ or Δ/α) on the trend of W-O switching frequency, we uniformly selected the *MTL*Δ/α strain for this assay. As shown in Figure 4 and Table 2, deletion of *CIT1* or *KGD1* completely blocked cell growth on Lee’s glucose (0.5%, w/v) + GlcNAc (1.5%, w/v) medium, indicating that these enzymes are essential for GlcNAc utilization. Although reduced cell growth was observed in the *sdh3/sdh3* mutants, no W-O switching was detected. In contrast, the WT and reconstituted strains exhibited comparable W-O switching frequency on both day 5 and day 10. These results suggest that the TCA enzymes are critical not only for GlcNAc utilization to support cell growth but also for generating metabolic signals that regulate W-O switching in *C. albicans*.

The WT (Δ/α) control, *cit1/cit1* (Δ/α), *kgd1/kgd1* (Δ/α), *sdh3/sdh3* (Δ/α) mutants, and the corresponding reconstituted strains were initially grown on Lee’s glucose medium at 25°C for 5 days. White cells of each strain were replated onto Lee’s glucose (0.5%) +GlcNAc (1.5%) medium and incubated at 25°C for 5 or 10 days. WT, SC5314; Wh, white; Op, opaque; Op-sec, opaque sectors. Scale bar for colonies, 1 mm. Scale bar for cells, 10 μm. The numerical data are shown in Table 2.

**Table 2.** White-to-opaque switching frequencies of the *MTL* homozygous WT, and deletion mutants of the key TCA cycle enzymes, and reconstituted strains on Lee’s glucose (0.5%) + GlcNAc (1.5%) medium at 25°C for 10 days.

Strain	Total colonies	% op colonies
WT (SC5314) (Δ/α)	378	51.8 ± 3.7
*cit1/cit1* (Δ/α)	/	/
*cit1/cit1*+*CIT1*p-*CIT1* (Δ/α)	490	24.1 ± 3.3
*kgd1/kgd1* (Δ/α)	/	/
*kgd1/kgd1*+*KGD1*p-*KGD1* (Δ/α)	761	27.6 ± 7.3
*sdh3/sdh3* (Δ/α)	860	<0.2
*sdh3/sdh3*+*SDH3*p-*SDH3* (Δ/α)	355	92.5 ± 3.8

### Roles of Mcu1 and TCA cycle enzymes in gut commensalism *in* *vivo*

**Figure 5.**
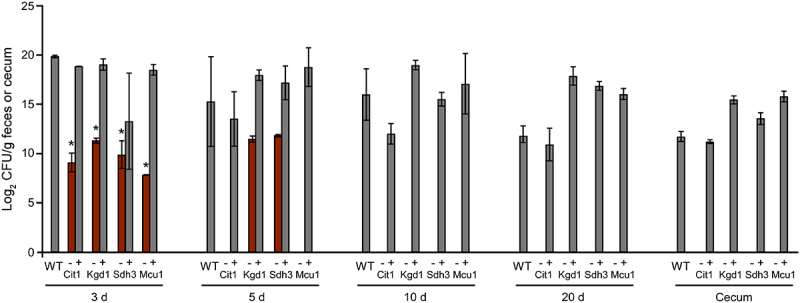
Mcu1 and TCA cycle enzymes are required for *C. albicans* commensal fitness.

Previous studies have shown that *C. albicans* modulates TCA cycle metabolism (e.g. by upregulating genes such as *CIT1* and *KGD1*) in response to changes in the host intestinal environment, coincident with shifts in the commensal-pathogenic equilibrium [21]. We further investigated whether Mcu1 and the TCA cycle enzymes contribute to normal commensal fitness of *C. albicans* in the host. Through a mouse oral gavage model, we assessed the effects of Mcu1 and TCA cycle enzymes on the colonization capacity of *C. albicans*. As shown in Figure 5, the WT and reconstituted strains exhibited comparable colonization levels over time. In striking contrast, all deletion mutants (including those lacking *CIT1*, *KGD1*, *SDH3*, and *MCU1*) exhibited severely impaired colonization. Only the *KGD1* and *SDH3* mutants remained detectable on day 5, and no mutants were observed by day 10. These results indicate that both Mcu1 and the TCA cycle enzymes are essential for sustaining gut commensalism of *C. albicans*.

Female BALB/c mice (6 weeks), pre-treated with antibiotics daily for 3 days, were orally inoculated with 200 μL of *C. albicans* suspension (approximately 2.5 × 10^8^ cells/mL). The WT control (**a**/α), *mcu1/mcu1* (**a**/α), *cit1/cit1* (**a**/α), *kgd1/kgd1* (**a**/α), and *sdh3/sdh3* (**a**/α) mutants, and the corresponding reconstituted strains were used. Fecal samples were collected and weighed on days 3, 5, 10, and 20 post-gavage. Cecum samples were collected after mice were euthanized. Each sample was homogenized and plated onto solid YPD medium. All plates were incubated at 30°C for 3 days for observation and colony counting. WT, SC5314. “-,” gene deletion mutants. “+,” reconstituted strains. Log_2_CFU/g represents the log_2_-transformed CFU per gram of fecal or cecal sample. Three replicates were carried out. Error bars represent the standard deviation (SD). *p < 0.05 (Student’s *t*-test, two‑tailed).

### Roles of Mcu1 in regulating Wor1, the master regulator of gut commensalism

**Figure 6.**
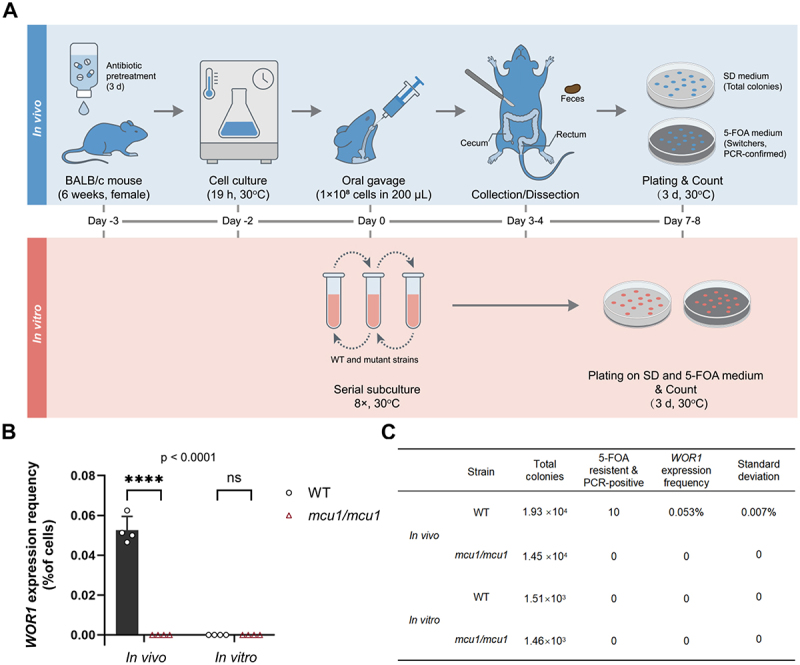
Mcu1 is required for the expression of *WOR1* in the commensal milieu.

Since Wor1 is a key regulator of the opaque and commensal GUT cell states, we therefore hypothesized that Mcu1 influences *in vitro* W-O switching and *in vivo* commensal-pathogenic switch by modulating *WOR1* expression. As expected, ectopic expression of *WOR1* in *MTL* homozygous *mcu1/mcu1* mutant resulted in 100% W-O switching *in vitro* (Figure S2). To examine its *in vivo* effect, we adopted an established reporter system from Pande et al. (2013) to examine *WOR1* expression *in vivo* in *MTL* heterozygous WT and *mcu1/mcu1* mutant strains [5]. Briefly, the *WOR1*_prom_-*FLP* strain (SN1020 **a**/α) contains a locus where the endogenous *WOR1* promoter is fused to the Flp recombinase gene and carries a copy of the selectable marker *URA3* [22,23]. This *URA3* marker is highly sensitive to 5-fluoroorotic acid (5-FOA) and is flanked by FRT recombination sites. Upon *WOR1* promoter activation, Flp recombinase is expressed, leading to *URA3* excision and conferring 5-FOA resistance. Therefore, we deleted the *MCU1* gene in the *WOR1*_prom_-*FLP* strain (yielding strain XM91 **a**/α) and examined *WOR1* expression changes after propagation in the murine commensal model or *in vitro* (Figure 6(A)). By quantifying colonies on 5-FOA selection medium vs non-selective SCD medium, we found that *in vivo* (commensal model), the WT *WOR1*_prom_-*FLP* strain exhibited normal induction of *WOR1* expression (frequency: 0.053 ± 0.007%), whereas no *WOR1* expression was detected in the *mcu1/mcu1 WOR1*_prom_-*FLP* mutant (Figure 6(B,C)). Under *in vitro* conditions, neither the WT nor *mcu1/mcu1* mutant induced *WOR1* expression. These findings indicate that the *WOR1* gene in *MTL* heterozygous strains is refractory to *in* *vitro* induction, but requires Mcu1 for its activation *in vivo*. Taken together, Mcu1 is required for host-specific induction of *WOR1* in *MTL*-heterozygous cells, thereby stabilizing the commensal state *in vivo*. Therefore, our findings position Mcu1 as a critical factor that links mitochondrial metabolic sensing to the transcriptional reprogramming necessary for commensal-pathogenic transition in *C. albicans*.

(A) Schematic representation of the estimation of *WOR1* expression both *in vivo* or *in vitro*. Female BALB/c mice (6 weeks) were pre-treated with antibiotics daily for 3 days, and then were orally inoculated with 200 μL of *C. albicans* suspension (approximately 5 × 10^8^ cells/mL). The WT+*WOR1*_prom_-*FLP* (SN1020, **a**/α) and *mcu1/mcu1*+*WOR1*_prom_-*FLP* (XM91, **a**/α) strains (approximately 1 × 10^8^ cells) were infected for 3 days in the murine commensal model or serially passaged for eight generations in liquid SCD medium. Each sample was homogenized and plated onto both solid 5-FOA selection medium and solid SCD nonselective medium. All plates were incubated at 30°C for 3 days for observation and colony counting. (B) Comparison of *WOR1* expression of the WT control (**a**/α) and *mcu1/mcu1* (**a**/α) cells grown in the commensal milieu or *in vitro*. The Flp-mediated excision of *URA3* was verified via 5-FOA selection and PCR identification of 5-FOA-resistant colonies. The frequency of cells expressing *WOR1* was represented as the ratio of (number of 5-FOA-resistant and PCR-positive colonies) vs (total number of colonies). Y-axis, percentages of cells expressing *WOR1*. Error bars indicate the standard deviation (SD) among the biological replicates. Statistical differences were determined by Student’s *t*-test, two-tailed. (C) Frequency of *URA3* excision *in vivo* and *in vitro*. The data are associated with panel B.

## Discussion

As a commensal and opportunistic pathogen, *C. albicans* poses a serious threat to human health, especially among immunocompromised patients. The molecular mechanisms underlying its transition from a commensal to a pathogenic state are still not fully elucidated. In this study, we reveal a mitochondrial-centered regulatory axis in which the mitochondrial protein Mcu1, in concert with key TCA cycle enzymes, utilizes GlcNAc to regulate phenotypic switching and gut commensalism. Mechanistically, Mcu1 orchestrates respiratory metabolism by modulating the expression of multiple oxidoreductases. Mcu1 and TCA cycle enzymes are both required for W-O switching *in vitro* and for host colonization *in vivo*. Mcu1 promotes gut commensalism by activating the master regulator Wor1 *in vivo* even in *MTL* heterozygous strains, thereby overriding the classical mating-type restriction. Collectively, our findings demonstrate that the Mcu1-TCA cycle axis integrates host-derived signals (e.g. GlcNAc) to govern the commensal-pathogenic transition in *C. albicans*. This work unveils a previously unrecognized model in which mitochondrial respiratory metabolism acts as a central regulator that balances commensalism and virulence in fungal pathogens.

We previously reported that Mcu1 is essential for GlcNAc utilization, filamentation and virulence in *C. albicans* [10]. Here, we found that Mcu1 is specifically required for GlcNAc-induced, but not CO_2_-induced, W-O switching and is indispensable for gut commensalism. GlcNAc is abundant in the intestinal environment, derived from host mucins and bacterial peptidoglycan, and serves as both a carbon source and a developmental signal [8]. Our results suggest that Mcu1 could be a precise sensor for GlcNAc in the intestinal niche, which might promote white-to-GUT phenotypic switching and commensalism likely by enabling *WOR1* expression. This model aligns with the concept that pathogens intimately link their metabolic state with commensal programs to optimize host adaptation. Supporting this notion, in *C. albicans*, the transcription factor Efg1, a major transcriptional factor of morphogenesis, also acts as a key repressor of commensalism by inhibiting lipid metabolism and the TCA cycle in the gut [21]. Beyond fungi, metabolic flexibility appears to be a conserved determinant for enteric colonization. For instance, the fitness of *E. coli* in the gut depends on its metabolic flexibility to respire under both aerobic and anaerobic conditions [24]. *Salmonella enterica* serovar Typhimurium often senses ethanolamine signaling to identify and adapt to specific host niches [25].

Proteomic analysis showed that a subset of oxidoreductases was downregulated and oxygen consumption was decreased in the *mcu1/mcu1* mutant, highlighting the critical role of Mcu1 in maintaining respiratory metabolism. This conclusion is further solidified by our subsequent finding that key TCA cycle enzymes (Cit1, Kgd1, Sdh3) are also essential for GlcNAc utilization and phenotypic switching. We propose that impairment of TCA cycle, whether through the loss of Mcu1 or direct deletion of its enzymes, disrupts the energy metabolism required for phenotypic switching and Wor1 expression, ultimately compromising host colonization. This is consistent with previous studies indicating that the TCA cycle governs transcriptional reprogramming by modulating cellular energy status, thereby regulating the yeast-to-hyphae switching [26,27].

A long-standing paradox in *C. albicans* biology is how the Wor1-dependent commensal GUT cell state can arise within the prevalent *MTL* heterozygous (**a**/α) population in the gut [5,28–30]. This study resolves this paradox by showing that host-derived signals, integrated through the Mcu1-TCA cycle axis, can override the canonical genetic repression of *WOR1* within the murine gut, thereby enabling commensalism. This highlights a critical limitation of *in vitro* models and underscores the power of host–pathogen interactions in reshaping fungal gene expression. It establishes Mcu1 as a key factor that “unlocks” the commensal program in the most clinically prevalent genetic background of *C. albicans*. However, the precise mechanism by which Mcu1 exerts this regulatory role remains to be fully elucidated.

We previously reported that Mcu1 homologs are primarily and specifically present in *Candida* species and are absent in humans, highlighting its potential as a fungus-specific therapeutic target [10]. Disruption of Mcu1 could potentially lock *C. albicans* in a nonpathogenic state or impair its commensal fitness. Unlike conventional fungicidal drugs, targeting the Mcu1-mediated commensal-pathogenic transition represents a novel anti-virulence strategy.

In summary, we have uncovered a mitochondrial-centered regulatory axis in which Mcu1 and the TCA cycle sense host-derived cues (e.g. GlcNAc), modulate mitochondrial respiratory metabolism, and thereby drive phenotypic switching and gut commensalism in *C. albicans*. This metabolic module is indispensable for both the establishment and maintenance of the commensal state within the mammalian gut. By linking mitochondrial metabolism to cell fate decisions within the host, our findings provide a key conceptual advance in understanding the metabolic basis of fungal commensalism and pathogenesis.

## Supplementary Material

Dataset S1.xlsx

Clean Copy of Supplementary Material - QVIR-2026-0084.R1.docx

## Data Availability

All datasets that support this study are included in the article/Supplementary Materials. The raw data and supplementary files are available in Figshare at https://doi.org/10.6084/m9.figshare.31152412. The proteomics data have been deposited in the PRIDE database at https://www.ebi.ac.uk/pride/archive/projects/PXD071309 under the accession code PXD071309.

## Open scholarship

This article has earned the Center for Open Science badge for Open Data. The data are openly accessible at https://www.ebi.ac.uk/pride/archive/projects/PXD071309.
