# Supplementary material for: The Mcu1 mitochondrial protein coordinates TCA cycle enzymes to modulate phenotypic switching and commensalism in Candida albicans
Source: Virulence. 2026 Aug 2;17(1):2711487. doi: 10.1080/21505594.2026.2711487 (PMC13432846; doi:10.1080/21505594.2026.2711487)
Supplement: Clean Copy of Supplementary Material - QVIR-2026-0084.R1.docx [file KVIR_A_2711487_SM2630.docx]

**Supplemental material**

**
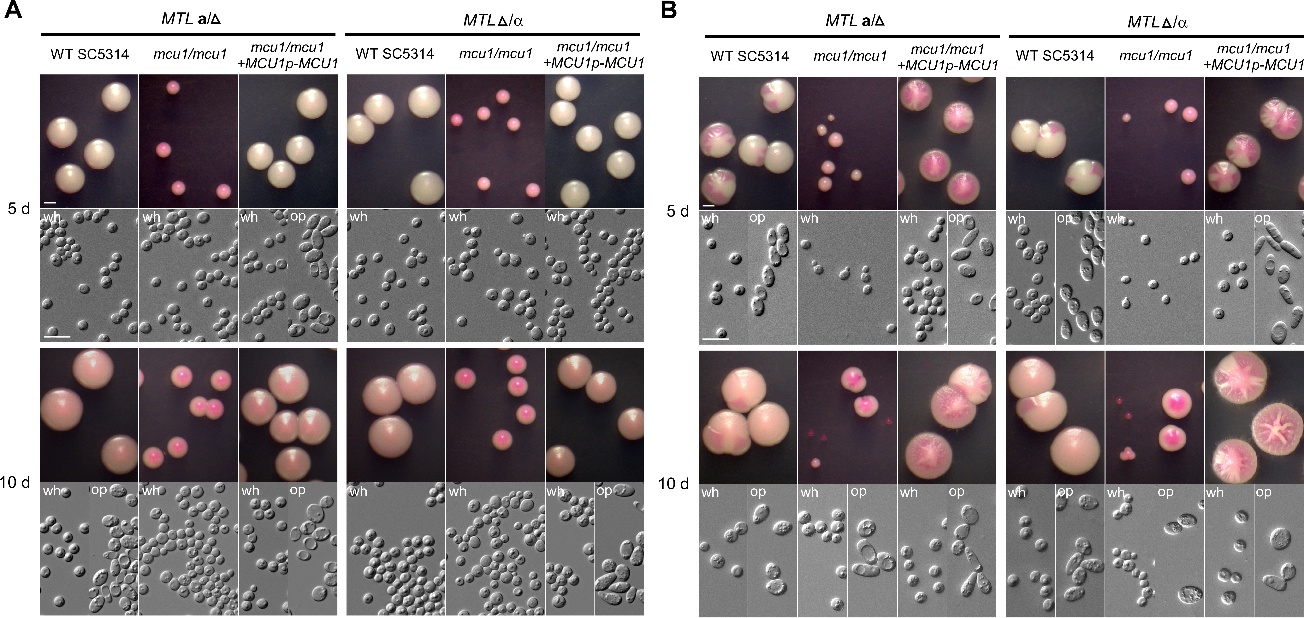
**

**Fig. S1. White-to-opaque switching of the *mcu1/mcu1* mutant on Lee’s glucose (2%) medium under air (A) or 5% CO_2_ (B) conditions.** Colony and cellular morphologies of the WT (**a**/Δ or Δ/α), *mcu1/mcu1* mutant (**a**/Δ or Δ/α), and reconstituted strain (**a**/Δ or Δ/α) grown on Lee’s glucose (2%). Strains were initially grown on Lee’s glucose (2%) medium at 25^o^C for 5 days. White cells of each strain were replated onto Lee’s glucose (2%) medium and incubated at 25^o^C for 5 or 10 days. WT, SC5314; Wh, white; Op, opaque. Scale bar for colonies, 1mm. Scale bar for cells, 10 μm. The numerical data are shown in **Table S3**.

**
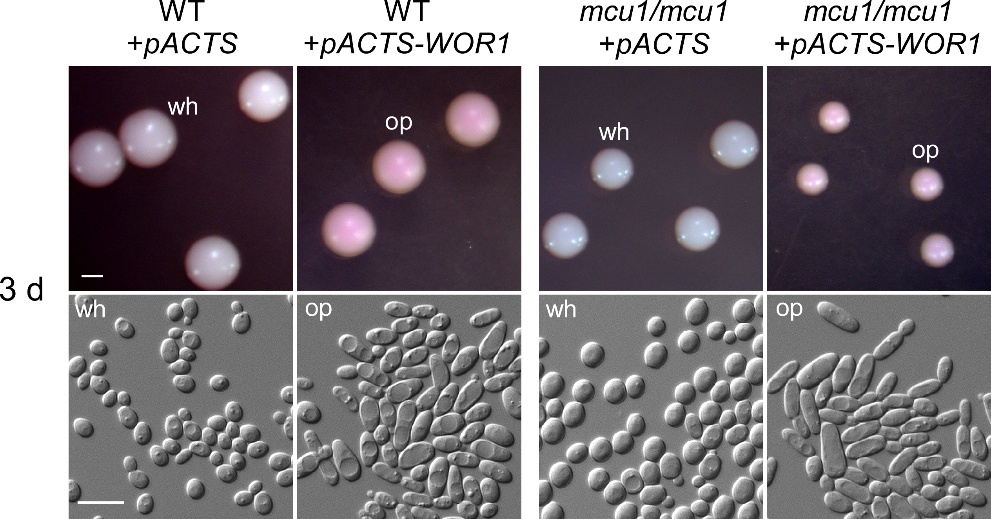
**

**Fig. S2. White-to-opaque switching of *WOR1*** **ectopic expression strain on YPD medium.** Colony and cellular morphologies of strains ectopically expressing *WOR1* in the WT (**a**/**a**) and *mcu1/mcu1* mutant (**a**/**a**) backgrounds, grown on YPD medium containing phloxine B at 25^o^C for 3 days. WT, GH1013; Wh, white; Op, opaque. Scale bar for colonies, 1mm. Scale bar for cells, 10 μm.

**Table S1 Strains used in this study.**

| **Strain name** | **Parent strain** | **Genotype** | **Reference** |
| --- | --- | --- | --- |
| SC5314 |  | Clinical isolate | [1] |
| SN152 | SC5314 | *ura3*:: λimm434::*URA3-IRO1*/*ura3*:: λimm434, *arg4*::hisG/*arg4*::hisG *his1*::hisG/*his1*::hisG *leu2*::hisG/*leu2*::hisG, *MTL***a**/α | [2] |
| SC5314 (**a**/Δ) | SC5314 | As SC5314, but *MTL***a**/*mtl*α::*FRT-SAT1-FRT* | This study |
| SC5314 (Δ/α) | SC5314 | As SC5314, but *mtl***a**/*MTL*α::*FRT-SAT1-FRT* | This study |
| BWP17 | SC5314 | *ura3*::imm434/*ura3*::imm434 *iro1/iro1*::imm434 *his1*::hisG/*his1*::hisG *arg4/arg4*, *MTL***a**/α | [3] |
| GH1013 | BWP17 | *MTL***a**/**a***, ura3::imm434/ura3::imm434, his1::hisG/his1::hisG, arg4::hisG/arg4::hisG* | [4] |
| SN78 | RM1000#2 | As RM1000#2, but *ura3*/*ura3* *iro1*/*iro1* *his1*/*his1 leu2/leu2*, *MTL***a**/α | [2] |
| SN1014 | SN78 | As SN78, but *wor1::FLP-SAT1/WOR1, leu2/leu2,*  *ura3/ura3, his1/his1, iro1/iro1* | [5] |
| SN1020 | SN1014 | As SN1014, but *wor1*::*FLP-SAT1/WOR1, leu2*::*FRT URA3-FRT/leu2, ura3 /ura3* | [5] |
| XM91 | SN1020 | As SN1020, but *mcu1*::*ARG4/mcu1*::*HIS1* | This study |
| *mcu1/mcu1* | BWP17 | As BWP17, but *mcu1*::*ARG4/mcu1*::*HIS1 ura3*::imm434/*URA3* | [6] |
| *mcu1/mcu1* (**a**/Δ) | *mcu1/mcu1* | As *mcu1/mcu1*, but *MTL***a**/*mtl*α::*FRT-SAT1-FRT* | This study |
| *mcu1/mcu1* (Δ/α) | *mcu1/mcu1* | As *mcu1/mcu1*, but *mtl***a**::*FRT-SAT1-FRT*/*MTL*α | This study |
| *mcu1*/*MCU1p-MCU1* | *mcu1/mcu1* | As *mcu1/mcu1,* but *MCU1p-MCU1-FRT* | [6] |
| *mcu1*/*MCU1p-MCU1* (**a**/Δ) | *mcu1*/*MCU1p-MCU1* | As *mcu1*/*MCU1p-MCU1*, but *MTL***a**/*mtl*α::*FRT-SAT1-FRT* | This study |
| *mcu1*/*MCU1p-MCU1* (Δ/α) | *mcu1*/*MCU1p-MCU1* | As *mcu1*/*MCU1p-MCU1*, but *mtl***a**::*FRT-SAT1-FRT*/*MTL*α | This study |
| *cit1*/*cit1* | SN152 | As SN152, but *cit1::ARG4/cit1::HIS1*  *leu2*::hisG/*LEU2* | [7] |
| *cit1*/*cit1* (Δ/α) | *cit1*/*cit1* | As *cit1*/*cit1*, but *mtl***a**::*FRT-SAT1-FRT /MTLα* | This study |
| *cit1*/*CIT1p-CIT1* | *cit1/cit1* | As *cit1/cit1,* but *CIT1p-CIT1-LEU2* | [7] |
| *cit1*/*CIT1p-CIT1* (Δ/α) | *cit1*/*CIT1p-CIT1* | As *cit1*/*CIT1p-CIT1*, but *mtl***a**::*FRT-SAT1-FRT /MTLα* | This study |
| *kgd1*/*kgd1* | SN152 | As SN152, but *kgd1::ARG4/kgd1::HIS1*  *leu2*::hisG/*LEU2* | [7] |
| *kgd1/kgd1* (Δ/α) | *kgd1/kgd1* | As *kgd1/kgd1,* but *mtl***a**::*FRT-SAT1-FRT /MTLα* | This study |
| *kgd1*/*KGD1p-KGD1* | *kgd1/kgd1* | As *kgd1/kgd1,* but *KGD1p-KGD1-LEU2* | [7] |
| *kgd1*/*KGD1p-KGD1* (Δ/α) | *kgd1/kgd1* | As *kgd1*/*KGD1p-KGD1*, but *mtl***a**::*FRT-SAT1-FRT /MTLα* | This study |
| *sdh3/sdh3* | SN152 | As SN152, but *sdh3::ARG4/sdh3::HIS1*  *leu2*::hisG/*LEU2* | [7] |
| *sdh3/sdh3* (Δ/α) | *sdh3/sdh3* | As *sdh3/sdh3,* but *mtl***a**::*FRT-SAT1-FRT /MTLα* | This study |
| *sdh3*/*SDH3p-SDH3* | *sdh3/sdh3* | As *sdh3/sdh3,* but *SDH3p-SDH3-LEU2* | [7] |
| *sdh3*/*SDH3p-SDH3* (Δ/α) | *sdh3*/*SDH3p-SDH3* | As *sdh3*/*SDH3p-SDH3*, but *mtl***a**::*FRT-SAT1-FRT /MTLα* | This study |
| GH1013+ *pACTS* | GH1013 | As GH1013, but *pACTS* | This study |
| *mcu1/mcu1* (**a**/**a**) | GH1013 | As GH1013, but *mcu1*::*ARG4/mcu1*::*HIS1* | This study |
| *mcu1/mcu1*+*pACTS* (**a**/**a**) | *mcu1/mcu1* (**a**/**a**) | As *mcu1/mcu1* (**a**/**a**), but *pACTS* | This study |
| GH1013+ *pACTS-WOR1* | GH1013 | As GH1013, but *pACTS-WOR1* | This study |
| *mcu1/mcu1*+*pACTS-WOR1* (**a**/**a**) | *mcu1/mcu1* (**a**/**a**) | As *mcu1/mcu1* (**a**/**a**), but *pACTS-WOR1* | This study |

**References:**

1. Fonzi WA, Irwin MY. Isogenic strain construction and gene mapping in *Candida albicans*. Genetics 134(3): 717-28 (1993).
2. Noble SM, Johnson AD. Strains and strategies for large-scale gene deletion studies of the diploid human fungal pathogen *Candida albicans*. Eukaryot Cell 4: 298-309 (2005).
3. Wilson RB, Davis D, Mitchell AP. Rapid hypothesis testing with *Candida albicans* through gene disruption with short homology regions. J Bacteriol 181(6): 1868-74 (1999).
4. Huang G, Srikantha T, Sahni N, Yi S, Soll DR. CO_2_ regulates white-to-opaque switching in *Candida albicans*. Curr Biol. 19: 330-4 (2009).
5. Pande K, Chen C, Noble SM. Passage through the mammalian gut triggers a phenotypic switch that promotes *Candida albicans* commensalism. Nat Genet 45(9): 1088-91 (2013).
6. Guan G, Wang H, Liang W, Cao C, Tao L, Naseem S, Konopka JB, Wang Y, Huang G. The mitochondrial protein Mcu1 plays important roles in carbon source utilization, filamentation, and virulence in *Candida albicans*. Fungal Genet Biol 81: 150-9 (2015).
7. Tao L, Zhang Y, Fan S, Nobile CJ, Guan G, Huang G. Integration of the tricarboxylic acid (TCA) cycle with cAMP signaling and Sfl2 pathways in the regulation of CO_2_ sensing and hyphal development in *Candida albicans*. PLoS Genet 13: e1006949 (2017).

**Table S2 Primers used in this study.**

| Name | Sequence (5’ to 3’) | Purpose |
| --- | --- | --- |
| *MTL***a**1 Fwd | TAAGAATGAAGACAACGAGG | *MTL***a**1 deletion confirmation |
| *MTL***a**1 Rev | TTATCATCATCCATCTGGTC |  |
| *MTL*α1 Fwd | TAAGAATGAAGACAACGAGG | *MTL*α1 deletion confirmation |
| *MTL*α1 Rev | TTATCATCATCCATCTGGTC |  |
| MCU1 up Fwd | CGAATCACAATCATCTCCAG | Knockout of *MCU1* |
| MCU1 up Rev | CACGGCGCGCCTAGCAGCGGGTTTGGCTTCTTGTAATAGTG |  |
| pSN marker Fwd | CCGCTGCTAGGCGCGCCGTGACCAGTGTGATGGATATCTGC |  |
| pSN marker Rev | GCAGGGATGCGGCCGCTGACAGCTCGGATCCACTAGTAACG |  |
| MCU1 down Fwd | GTCAGCGGCCGCATCCCTGCCAAGTGGAGTAACGAGTATG |  |
| MCU1 down Rev | CAACCTTCCATTTATCCGTCC |  |
| HIS1-5-detect | ATTAGATACGTTGGTGGTTC | Verification of the *mcu1/mcu1* mutant |
| HIS1-3-detect | AACACAACTGCACAATCTGG |  |
| ARG4-5-detect | TGCATTGACTACAGTGGAAC |  |
| ARG4-3-detect | ATCATGCCATTCTTGTCTG |  |
| MCU1 check Fwd | ATCAGAAGTAAGTCCACAGTAG |  |
| MCU1 check Rev | GGATCTTGTTTGTGGTTGAAG |  |
| MCU1 ORF Fwd | TACCAGGCCAAGAACCAAGTG |  |
| MCU1 ORF Rev | CATCTCTAACATTTCTAACCAC |  |
| SNO509 | GTTGTGATTTTGCTATTCCGGCGCT | *URA3* excision confirmation |
| SNO840 | TCTCTCCGAATGAAGAGCC |  |
| SNO1535 | AGAAAGAAAGAGAGAGAGGGAACG | *WOR1p-FLP* strain confirmation |
| SNO1536 | CTGTTCCGTTATGTGTAATCATCC |  |
| SNO1537 | CGCCTAACATATGTGAAGTGTGA |  |
| SNO1354 | CGTTCAGATATTCATACATCCACCT |  |
| WOR1 OE Fwd | ATATGATATCATGTCTAATTCAAGTATAGTCCCT | Ectopic expression of *WOR1* |
| WOR1 OE Rev | ATATAAGCTTATGATTTTCTGGATTTCCGTG |  |

**Table S3 White-to-opaque switching frequencies of *MTL* homozygous (a/Δ or** **Δ/α) WT, *mcu1/mcu1* mutant, and reconstituted strains on Lee’s glucose (2%) medium in air and 5% CO_2_.**

| **Strain** | **Air** | |  | **5% CO_2_** | |
| --- | --- | --- | --- | --- | --- |
|  | **Total colonies** | **% op colonies** |  | **Total colonies** | **% op colonies** |
| WT (SC5314) (**a**/Δ) | 623 | 0.2 ± 0.4 |  | 680 | 98.1 ± 0.9 |
| *mcu1/mcu1* (**a**/Δ) | 617 | < 0.2 |  | 421 | 93.8 ± 4.8 |
| *mcu1/mcu1*+*MCU1*p-*MCU1* (**a**/Δ) | 734 | 0.1 ± 0.3 |  | 744 | 100 ± 0.0 |
| WT (SC5314) (Δ/α) | 568 | < 0.2 |  | 375 | 96.5 ± 2.0 |
| *mcu1/mcu1* (Δ/α) | 657 | < 0.2 |  | 386 | 94.3 ± 1.9 |
| *mcu1/mcu1*+*MCU1*p-*MCU1* (Δ/α) | 564 | 0.7 ± 0.8 |  | 839 | 100 ± 0.0 |

**Dataset S1 Protein expression profiles of the WT (a/α) and *mcu1/mcu1* (a/α) mutant strains grown in YPD liquid medium at 30^o^C.**
